# Supplementary material for: COZOID: contact zone identifier for visual analysis of protein-protein interactions
Source: BMC Bioinformatics. 2018 Apr 6;19:125. doi: 10.1186/s12859-018-2113-6 (PMC5889581; doi:10.1186/s12859-018-2113-6)
Supplement: Supplementary file 2 — Software build. Executable binary file of the software tool. (ZIP 118,784 kb) [file 12859_2018_2113_MOESM2_ESM.zip › cozoid/java/docs/beansbinding-1.2.1-doc.zip!/overview-summary.html]

Overview (Beans Binding)


---


|  |  |  |
| --- | --- | --- |
| **Overview** \| Package \| Class \| Use \| **Tree** \| **Deprecated** \| **Index** \| **Help** | |  |
| PREV   NEXT | **FRAMES**    **NO FRAMES**     **All Classes** |  |


---

| **Packages** | |
| --- | --- |
| **org.jdesktop.beansbinding** | Provides support for defining properties and creating bindings between sets of two properties. |
| **org.jdesktop.beansbinding.ext** | Provides support for customizing Beans Binding, and for determining the synthetic properties available for binding. |
| **org.jdesktop.el** | Customized version of EL for Beans Binding; not for general use. |
| **org.jdesktop.el.impl** | Customized version of EL for Beans Binding; not for general use. |
| **org.jdesktop.el.impl.lang** | Customized version of EL for Beans Binding; not for general use. |
| **org.jdesktop.el.impl.parser** | Customized version of EL for Beans Binding; not for general use. |
| **org.jdesktop.el.impl.util** | Customized version of EL for Beans Binding; not for general use. |
| **org.jdesktop.observablecollections** | Provides support for observing changes to collection classes. |
| **org.jdesktop.swingbinding** | Provides support for binding to complex Swing components, and documentation on the interesting Swing properties to bind to. |
| **org.jdesktop.swingbinding.adapters** | Private implementation details for Swing binding. |
| **org.jdesktop.swingbinding.impl** | Private implementation details for Swing binding. |

---


|  |  |  |
| --- | --- | --- |
| **Overview** \| Package \| Class \| Use \| **Tree** \| **Deprecated** \| **Index** \| **Help** | |  |
| PREV   NEXT | **FRAMES**    **NO FRAMES**     **All Classes** |  |


---
